# Variation in the main health-promoting compounds and antioxidant activity of different organs of Wasabi (Eutrema *japonicum*) from two producing areas

**DOI:** 10.3389/fpls.2022.1043378

**Published:** 2022-10-26

**Authors:** Hongmei Di, Cexian Cui, Pengcheng Fang, Junying Ma, Maolin He, Mengyao Li, Wei Lu, Fen Zhang, Yangxia Zheng

**Affiliations:** College of Horticulture, Sichuan Agricultural University, Chengdu, China

**Keywords:** Wasabi (Eutrema *japonicum*), organs, producing area, antioxidants, glucosinolates, glucosinolate breakdown products

## Abstract

Wasabi (Eutrema *japonicum*), also known as Japanese horseradish, is a perennial herb widely used in Japanese cuisine for its special flavour. The health-promoting phytochemicals and antioxidant capacity of four organs (leaf, petiole, rhizome, and root) of two cultivars (Chuankui–1 and Chuankui–2) of wasabi from two producing areas, Leibo and Guangyuan in Sichuan Province, China, were investigated in this study. The results showed that leaves were rich in pigments, soluble protein, ascorbic acid, and total phenolics and had the highest antioxidant capacity. Soluble sugars were highest in the petioles and were 1.1- to 5-fold higher than those in the other three organs. Glucosinolates and glucosinolate breakdown products (GBPs) were the most abundant in rhizomes, and their maximum values were 271.61 mmol kg^-1^ DW and 249.78 mmol kg^-1^ DW, respectively. The rhizomes of Chuankui–1 in Leibo and the leaves of Chuankui–1 in Guangyuan were superior in terms of glucosinolates and GBPs. These findings provide new insights that will aid the use of wasabi cultivars; they also have implications for the environmental characteristics needed to obtain better quality wasabi products. In the future, metabolome and transcriptome can be used to analyze the potential mechanism of differences among typical varieties, origins and parts.

## Introduction

Wasabi (Eutrema *japonicum*), otherwise known as Japanese horseradish, is a perennial herb (Depree et al., 1999). It is widely planted in Japan because it is a common ingredient in traditional Japanese dishes; wasabi is also cultivated in New Zealand (Sultana et al., 2003a; Qiu et al., 2019). Wasabi rhizomes are green, have a columnar shape and white pith, and are edible. It takes 1.5 to 2 years to harvest wasabi (Kinae et al., 2000). The abundant glucosinolates and glucosinolate breakdown products (GBPs) in rhizomes are also the main flavour substances and functional components (Hao et al., 2016; Qiu et al., 2019). Glucosinolates are secondary metabolites unique to the Cruciferae family. Myrosinase hydrolyses glucosinolates into various GBPs including isothiocyanates (ITCs), nitriles (CNs), and epithionitriles (EPNs) when plant tissues are damaged, and cells are destroyed (such as when rhizomes are ground into paste). In the absence of epithiospecifier proteins (ESPs), ITCs are spontaneously formed; otherwise EPNs or CNs are generated (Zeng et al., 2021). Wasabi is also rich in ascorbic acid, phenolics, and other health-promoting phytochemicals (Shin et al., 2004).

The rhizomes are the main edible parts of wasabi. Traditionally, they are sold in bundles with the leaves cut off (Hodge, 1974). Previous studies examining the content of ITCs in rhizomes have compared the yield of ITCs in the rhizomes of wasabi grown in soil and water (Sultana et al., 2003b) and the ITC content in the rhizomes of flowering and non-flowering wasabi plants (Sultana et al., 2003a). However, the demand for leaves and petioles is increasing; for example, the leaves and petioles are often consumed in pickled form and eaten with rice. A previous study examining the content of total phenolics, total flavonoids, ITCs, and antioxidant capacity in different organs of wasabi has shown that the value of wasabi leaves is underappreciated (Shin et al., 2014). Few comparative studies on wasabi organs have been conducted. It is very necessary to study the differences of health-promoting substances in different organs, which can not only improve the utilization of previously neglected organs to improve the overall economic value of products, but also utilize the rich nutrients from different organs, respectively. For example, sweet potato (Ipomoea *batata* L.) as an alternate food crop, its edible organ is usually root and nutrient of concern is mainly starch, while studies have found that its leaves are richer in phenolic substances, indicating that leaves of sweet potato merit inclusion in diets as leaf vegetables (Jung et al., 2011).

The substantial increase in demand for wasabi paste and downstream products, such as suancai, salads, and snacks, has increased their economic value and the area of wasabi cultivation. However, wasabi grows optimally in cool and humid environments; it can thus only be planted at elevations from 1500 to 2600 m under specific growth conditions (Hodge, 1974). Leibo and Guangyuan in Sichuan Province, China, are the two main areas of Sichuan wasabi production, and the climate of these two sites differs. No studies to date have compared the phytochemicals of wasabi products derived from these regions. Chuankui–1 (C1) and Chuankui–2 (C2) are typical cultivars that have been cultivated for a long time and have a wide range of cultivation. Here, we characterized the content of pigments, soluble protein, soluble sugars, ascorbic acid, total phenolics, glucosinolates, GBPs, and the antioxidant capacity in different organs of these two wasabi cultivars in Leibo and Guangyuan. Correlation analysis, principal components analysis, and variance analysis were also performed.

## Materials and methods

### Plant materials

**Figure 1:**
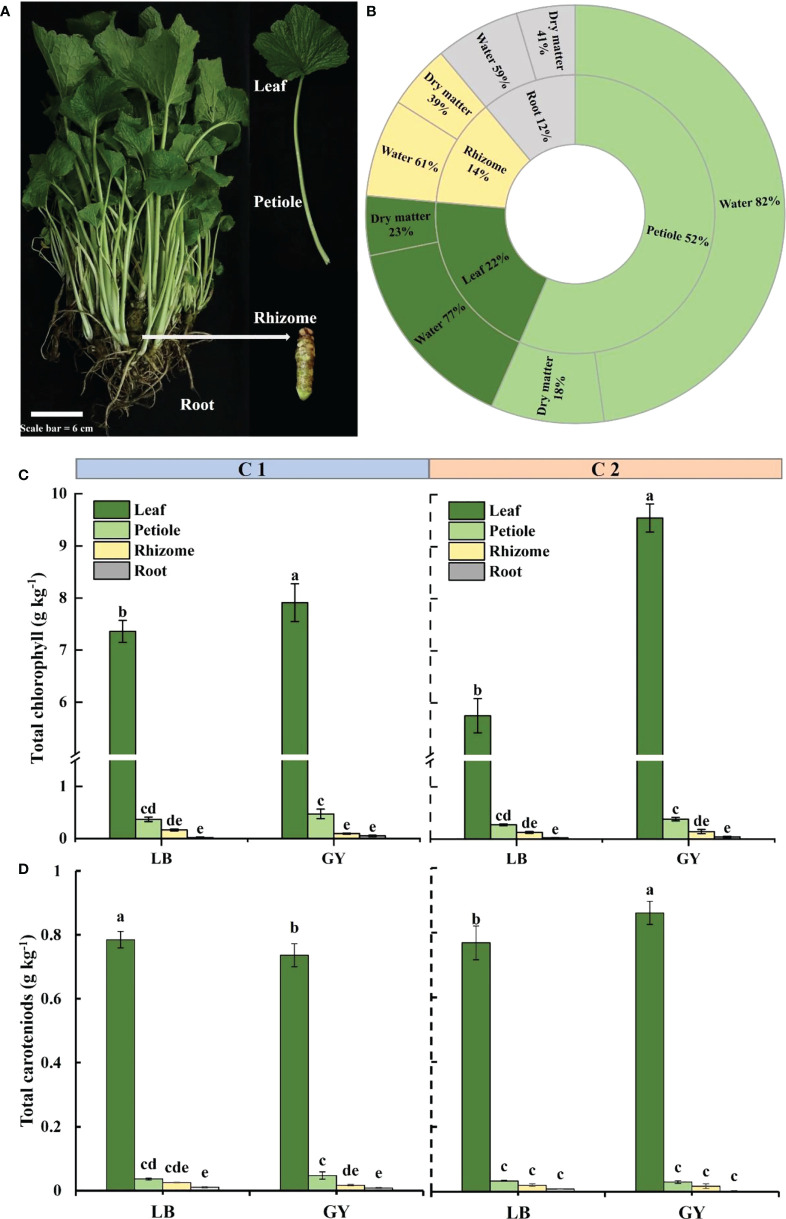
Visual appearance **(A)** and distribution and percentage of dry matter and water content in different organs **(B)** of wasabi. Total chlorophyll content **(C)** and carotenoids content **(D)** in different organs of two cultivars of wasabi from different producing areas. LB, Leibo; GY, Guangyuan Different lowercase letters indicate significant difference between different producing areas and organs of wasabi samples at 0.05 level.

Two cultivars of wasabi (Eutrema *japonicum*), C1 and C2, were grown in Gudui Township, Leibo City (28°360´N, 103°100´E, 2300 m altitude) and Tianxing Town, Guangyuan (32°527´N, 106°232´E, 1400 m altitude) Sichuan Province, China. The average summer temperatures in the two areas are 13 and 23°C respectively; the average winter temperatures are 0.1 and -6°C respectively; the annual rainfall is 813.6 and 1300 mm respectively. The soil is loose and fertile, with a pH of 5.9 to 7.0, with clean and sufficient irrigation water. The seeds of wasabi were sown in March 2020, and 6 months later, plants were transferred to the field at the four-leaf stage. In March of the following year, apply 750 kg of potassium sulfate compound fertilizer per hectare. At the end of September and in March of the following year, continue to apply potassium sulfate compound fertilizer, 450 kg and 750 kg per hectare respectively. Water was given as needed throughout the growth period. Carbendazim was sprayed to prevent soft rot. Materials were harvested uniformly in May 2022. Four groups (C1-LB, C1-GY, C2-LB, and C2-GY) of 60 wasabi plants were harvested at the same time in the morning and quickly transported to the laboratory. Fifteen plants from each group were divided into three replicate groups for experiments. In the laboratory, the surfaces of wasabi plants were washed, dried, sorted into four organs (leaves, petioles, rhizomes, and roots) (**Figure 1A**), and then weighed. The dry matter content and water content were determined for several samples (20 ± 2 g) of each organ. The remaining samples were mixed for the determination of health-promoting phytochemicals. The samples were then lyophilized in a freeze-dryer and stored at −20°C until further analysis.

### Dry matter and water content

Samples (20 g) were placed in an oven at 80°C until a constant weight was achieved to determine the dry matter content of each organ. The dry matter content (%) was calculated using the formula (W_F_ − W_D_)/W_F_ × 100, where W_F_ is the fresh weight, and W_D_ is the dry weight.

The water content (%) was calculated from the dry matter content: 100 – (W_F_ − W_D_)/W_F_ × 100 (Zheng et al., 2022).

### Chlorophyll and carotenoid content

Frozen powder (50 mg) of leaves and other samples (300 mg) were ground and extracted with 10 mL 96.0% ethanol. After centrifugation at 4000 g for 5 min, the supernatant was collected and total chlorophyll content was measured by reading the absorbance at 665 nm and 649 nm with a spectrophotometer (Sun et al., 2018).

Frozen powder (50 mg) was extracted with 10 mL of a mixture of acetone and petroleum ether (1:1, v/v) and petroleum ether was used for constant volume. The content of total carotenoid was measured by reading the absorbance at 451 nm with spectrophotometer. Result of chlorophyll and carotenoids content was expressed as g kg^−1^ of dry weight (Sun et al., 2018).

### Soluble protein content

The sample powder was soaked in 10 mL of double distilled H_2_O, centrifuged for 5 min at 4000 g and 1 mL transferred to a polypropylene tube. Subsequently, Coomassie brilliant blue G250 was combined with 1 mL of supernatant. The absorbance was measured at 595 nm within 20 min after the reaction. Result of soluble protein content was expressed as g kg^−1^ of dry weight (Sun et al., 2018).

### Soluble sugar content

The sample powder was extracted in 10 mL of double distilled H_2_O for 20 min at 90°C, centrifuged at 4000 g for 5 min. A combination of 1 mL of sample extract, 0.5 mL anthrone-ethylacetate reagent, and 5 mL concentrated sulfuric acid was homogenized and boiled for 5 min, and then cooled rapidly. The absorbance of the reaction mixtures was measured at 630 nm using a spectrophotometer. Result of soluble sugar content was expressed as g kg^−1^ of dry weight (Sun et al., 2018).

### Ascorbic acid content

The sample powder was extracted with 1.0% oxalic acid, and then centrifuged. Each sample was filtered through a 0.45 μm cellulose acetate filter, and analyzed by high performance liquid chromatography (HPLC). The amount of ascorbic acid was calculated from absorbance values at 243 nm. Result of ascorbic acid content was expressed as g kg^−1^ of dry weight (Sun et al., 2018).

### Total phenolics content

Total phenolics were homogenized for 1 min and extracted with 10 mL of 50% ethanol. The ethanol extract was mixed with Folin-Ciocalteu reagent after incubation in the dark at room temperature for 24 h, and saturated sodium carbonate was added after 3 min. The absorbance was measured at 760 nm with the spectrophotometer. Result of total phenolics content was expressed as g kg^−1^ of dry weight (Sun et al., 2018).

### Ferric reducing antioxidant power

The extracted samples were added to the FRAP working solution incubated at 37°C. The absorbance was then recorded at 593 nm using a spectrophotometer after the mixture had been incubated in at 37°C for 10 min, and then the value was calculated and the result was expressed as mmol kg^−1^ of dry weight (Sun et al., 2018).

### 2,2-azinobis (3-ethyl-benzothiazoline-6-sulfonic acid) assay

An aliquot of 300 μL of each extracted sample was added to 3 mL of ABTS^+^ solution. The absorbance was measured spectrophotometrically at 734 nm after exactly 2 h, and then the value was calculated (Sun et al., 2018).

### Glucosinolate composition and content

Freeze-dried samples (100 mg) were boiled in 5 mL water for 10 min. The supernatant was collected and applied to a DEAE-Sephadex A-25 column. The glucosinolates were converted into their desulpho analogues following aryl sulphatase, then the desulphoglucosinolates were eluted, and analyzed by HPLC. Result of glucosinolate content was expressed as mmol kg^−1^ of dry weight (Sun et al., 2018).

### Glucosinolate breakdown products composition and content

Freeze-dried samples (100 mg) were ground in 2 mL of water in the presence of 0.2 μmol of the internal standard benzonitrile. The solution was incubated at 25°C for 2 h, and dichloromethane (5 mL) was added, vortexed, and then centrifuged. The supernatant (1 mL) was transferred and analyzed using an Agilent 7890A Series GC System with an HP5 column (30 m × 0.25 mm × 0.25 μm). Result of GBPs content was expressed as mmol kg^−1^ of dry weight (Zhang et al., 2022).

### Data analysis

Data were analyzed using two-way analysis of variance. The means were compared through the least significant difference (LSD) test at a significance level of 0.05. Hierarchical clustering analysis and heatmap were generated using Origin 2022. Principal component analysis (PCA)and orthogonal partial least squares-discriminant analysis (OPLS-DA) were performed using SIMCA 14.1 software (Sun et al., 2021).

## Results

### Weight, dry matter content, and water content

The petioles made up most of the weight of the plants (52%), followed by the leaves, rhizomes, and roots (**Figure 1B**). The dry matter content was higher in the roots and rhizomes (41% and 39%, respectively), and the dry matter content in the leaves and petioles was low (approximately 20%). The opposite pattern was observed concerning the water content; the water content was highest in petioles and lowest in root fraction.

### Chlorophyll and carotenoids content

Regardless of the cultivar and producing area, the chlorophyll content was highest in the leaves, followed by the petiole, and the chlorophyll content was low in the rhizomes and roots fraction (**Figure 1C**). In C1, the chlorophyll content was significantly higher in the leaves of plants grown in Guangyuan than in Leibo. The difference was higher in C2 (as high as 1.7-fold). The same pattern was observed for the carotenoids content, which was much higher in the leaves than in the other organs (**Figure 1D**).

### Soluble protein and sugars content

**Figure 2:**
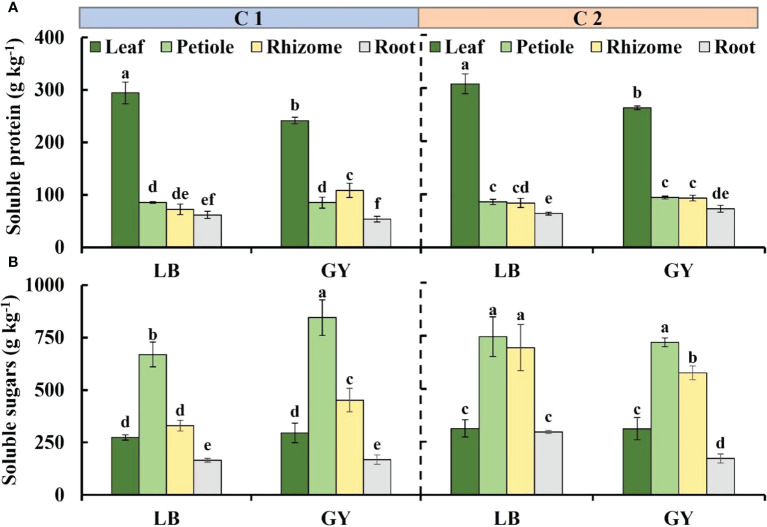
Soluble protein content **(A)** and soluble sugars content **(B)** in different organs of two cultivars of wasabi from different producing areas. LB, Leibo; GY, Guangyuan Different lowercase letters indicate significant difference between different producing areas and organs of wasabi samples at 0.05 level.

The content of soluble protein was much higher in wasabi leaves than in other organs, and it was lowest in the roots (**Figure 2A**). There was no difference in the content of soluble protein between the petioles and rhizomes in all groups, with the exception of the soluble protein content in the rhizomes of C1 grown in Guangyuan, which was higher than that in the petioles. The soluble protein content in the leaves of both cultivars of wasabi grown in Leibo was 1.2-fold higher than that of the leaves of wasabi grown in Guangyuan.

The distribution of soluble sugar content was petioles, rhizomes, leaves, roots from high to low except for no difference between petioles and rhizomes as well as leaves and roots of C2 grown in Leibo, respectively (**Figure 2B**). In C1, the soluble sugar content was higher in the petioles and rhizomes of plants grown in Guangyuan than in plants grown in Leibo. In C2, the regional difference is no longer, and the content in the rhizomes of wasabi grown in Leibo was higher than that in Guangyuan, even similar to that in the leaves.

### Ascorbic acid and total phenolics content

**Figure 3:**
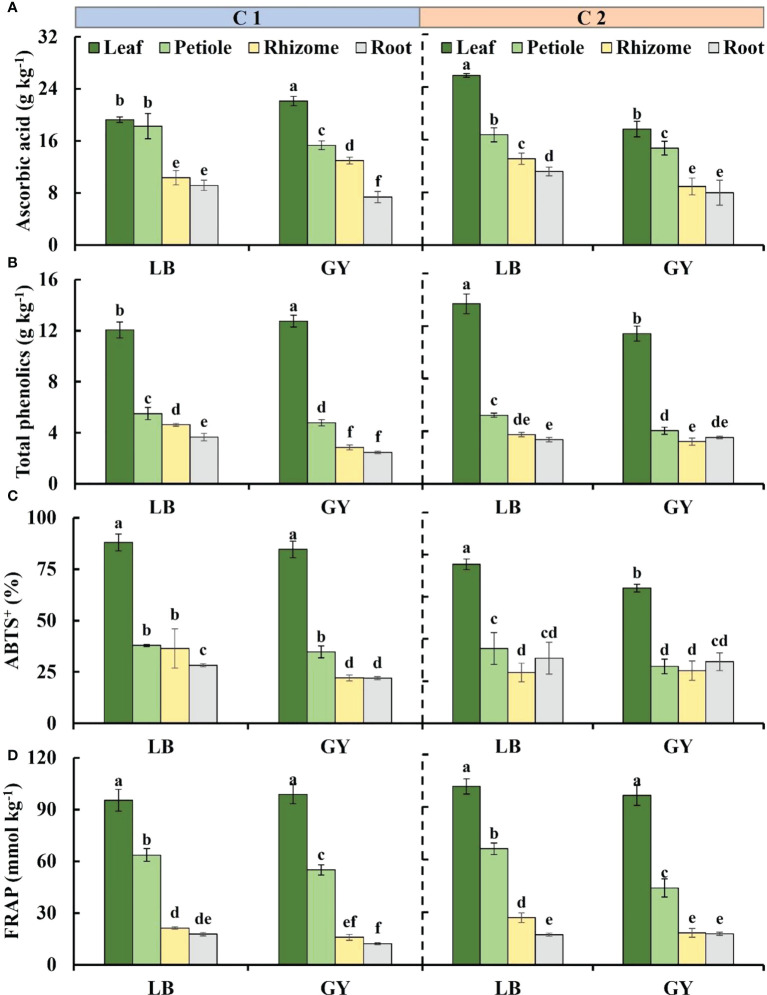
Main antioxidants content and antioxidant capacity levels in different organs of two cultivars of wasabi from different producing area. LB, Leibo; GY, Guangyuan. **(A)**: ascorbic acid; **(B)**: total phenolics; **(C)**: ABTS^+^; **(D)** FRAP Different lowercase letters indicate significant difference between different producing areas and organs of wasabi samples at 0.05 level.

The distribution of ascorbic acid content from high to low was leaves, petioles, rhizomes, and roots. There was no difference between the leaves and petioles, rhizomes and roots of C1 grown in Leibo, respectively; compared with the plants grown in Leibo, the content in leaves and rhizomes grown in Guangyuan was higher and the content in petioles and roots was lower. In C2, the ascorbic acid content in each organ was higher in plants grown in Leibo than in plants grown in Guangyuan (**Figure 3A**).

The total phenolics content was highest in the leaves, and it reached 13.7 g kg^-1^ in C2 grown in Guangyuan, followed by the petioles, and the content in the rhizomes and roots was lower than 5 g kg^-1^, which was almost one-third of that in the leaves. In C2, the total phenolics content in the leaves and petioles of plants grown in Leibo was higher, but there was no significant difference in the rhizomes and roots. The content in the leaves of C1 grown in Leibo was lower than that in Guangyuan, but the other three organs were higher in Leibo (**Figure 3B**).

### Antioxidant capacity

The ABTS^+^ values was much higher in the leaves than in the other organs, and the difference ranged from 2.0- to 3.8-fold. In C1, the ABTS levels of the rhizomes and roots of the plants grown in Leibo were higher than those of the corresponding organs in Guangyuan. In C2, the level was higher in the petioles of Leibo than that in Guangyuan.(**Figure 3C**).

The FRAP level was highest in the leaves, and it was as high as 101.8 g kg^-1^ in C2 grown in Leibo. The FRAP level was 45% of that in the leaves and 2.4-fold higher than that in the rhizomes and roots. Regardless of cultivar, the FRAP level of wasabi grown in Leibo was higher than that of wasabi grown in Guangyuan (**Figure 3D**).

### Glucosinolates content

**Figure 4:**
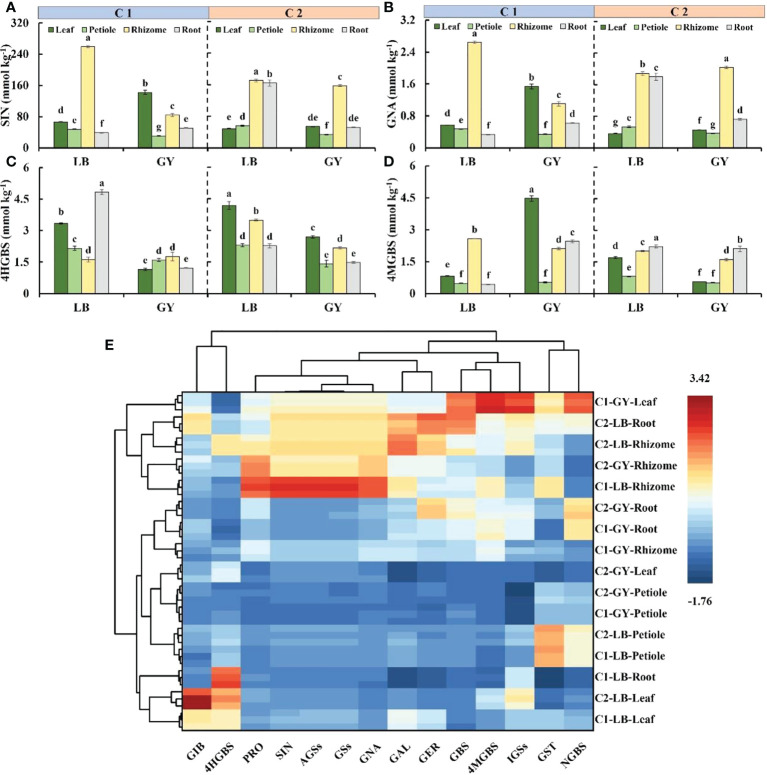
Main glucosinolate content **(A–D)** and hierarchical clustering analysis and heatmap **(E)** of all glucosinolates in different organs of two cultivars of wasabi from different producing area. C1, Chuankui–1; C2, Chuankui–2; LB, Leibo; GY, Guangyuan. **(A)**: sinigrin, SIN; **(B)**: gluconapin, GNA; **(C)**: 4-hydroxy glucobrassicin, 4HGBS; **(D)**: 4-methoxyglucobrassicin, 4MGBS Different lowercase letters indicate significant difference between different producing areas and organs of wasabi samples at 0.05 level.

Sinigrin was the most abundant glucosinolate in wasabi, accounting for more than 84% of the total glucosinolates and 94% of the aliphatic glucosinolates (**Supplementary Table S1**). In C1, the sinigrin content was highest in the rhizomes of wasabi grown in Leibo, reaching 259.1 mmol kg^-1^, followed by the leaves (66.3 mmol kg^-1^); the sinigrin content in the petioles and roots was lower, and it was only 17% of the content in the rhizomes on average. The sinigrin content in the leaves was highest in C1 grown in Guangyuan, followed by the rhizomes, and it was lowest in the petioles, which was 22% of that in the leaves. In C2, the sinigrin content was higher in the rhizomes and roots of wasabi grown in Leibo, with an average of 167.5 mmol kg^-1^, which was 3.0- and 3.4-fold higher than that in the petioles and leaves, respectively. The sinigrin content was high in the rhizomes of plants grown in Guangyuan, which was approximately 4.7-fold higher than that in the petioles, which had the lowest sinigrin content. In addition, the sinigrin content was slightly higher in the rhizomes of C2 grown in Leibo than that in Guangyuan, and the sinigrin content was much higher in the roots in C2 grown in Leibo than in C2 grown in Guangyuan (**Figure 4A**). The same pattern was observed for gluconapin, which was the second most abundant aliphatic glucosinolate. The rhizome gluconapin content was highest in C1 grown in Leibo, which was 2.6 mmol kg^-1^; in C1 grown in Guangyuan, the gluconapin content was highest in the leaves (only 1.5 mmol kg^-1^). In C2, the content of gluconapin was high in the rhizomes and roots of wasabi grown in Leibo; the highest gluconapin content was observed in the rhizomes of plants grown in Guangyuan (**Figure 4B**).

4-Hydroxyglucobrassicin and 4-methoxyglucobrassicin were abundant indolic glucosinolates, which jointly accounted for 61% to 95% of total indolic glucosinolates. The content of 4-hydroxyglucobrassicin was highest in the roots of C1 grown in Leibo (4.8 mmol kg^-1^), and the content of 4-hydroxyglucobrassicin in the roots was 3.0-fold higher than that in the rhizomes, which had the lowest content of 4-hydroxyglucobrassicin. The overall content of C1 grown in Guangyuan was relatively low, and the trend was opposite. C2, regardless of producing area, has the highest content in the leaves, followed by the rhizomes, and the lowest in the petioles and roots. The overall 4-hydroxyglucobrrassicin content was higher in C2 grown in Leibo than in plants grown in Guangyuan (**Figure 4C**). The 4-methoxyglucobrassicin content was highest in the rhizomes of C1 grown in Leibo (2.6 mmol kg^-1^), followed by the leaves. The content of 4-methoxyglucobrassicin in C1 grown in Guangyuan was highest in the leaves (as high as 4.5 mmol kg^-1^) followed by the roots and rhizomes; the content of 4-methoxyglucobrassicin in C1 was lowest in the petioles (0.4 mmol kg^-1^). Differences between the organs of C2 grown in the two producing areas were similar; the 4-methoxyglucobrassicin content was highest in the roots and lowest in the petioles (**Figure 4D**).

Gluconasturtiin was the only aromatic glucosinolate detected in wasabi. In C1, the content was highest in petioles grown in Leibo while in Guangyuan the content was highest in leaves; The lowest content was in roots. In C2, the highest content was in the petioles grown while leaves had the lowest content in the two producing areas.

The abundances of all glucosinolates are shown by organ in the form of a heatmap in **Figure 4E**, and detailed content information is shown in **Supplementary Table S1**. Variation in the content of glucoiberin among organs was similar to that of 4-hydroxyglucobrassicin; its content was higher in the leaves of the two cultivars of wasabi grown in Leibo than in plants grown in Guangyuan. Variation in the content of progoitrin among organs was similar to that of sinigrin and gluconapin; the content of progoitrin was highest in the rhizomes and leaves of C1 grown in Guangyuan. The content of glucoalysin and glucoerucin was high in the rhizomes and roots. Variation in the content of glucobrassicin was similar to that of 4-methoxyglucobrassicin; the content of glucobrassicin was highest in the leaves of C1 grown in Guangyuan. Clustering of gluconasturtiin and neoglucobrassicin, stemmed from their high content in the petioles of wasabi grown in Leibo.

### Glucosinolate breakdown products content

**Figure 5:**
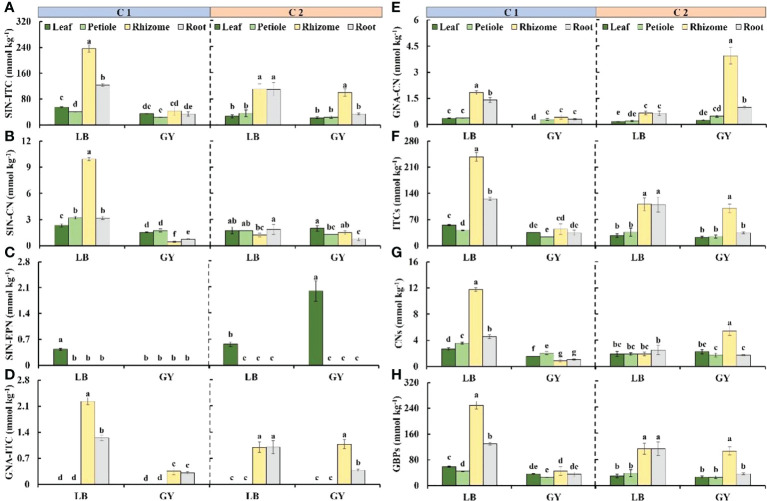
Glucosinolate breakdown products content in different organs of two cultivars of wasabi from different producing area. LB, Leibo; GY, Guangyuan. **(A)**: SIN-ITC, 2-propenyl isothiocyanate; **(B)**: SIN-CN, 3-butenenitrile; **(C)**: GNA-ITC, 3-butenyl isothiocyanate; **(D)**: GNA-CN, 4-pentenenitrile; **(E)**: SIN-EPN, 3,4-epithiobutanenitrile; **(F)**: ITCs, isothiocyanates; **(G)**: CNs, nitriles; **(H)**: GBPs, glucosinolate breakdown products Different lowercase letters indicate significant difference between different producing areas and organs of wasabi samples at 0.05 level.

Five GBPs were detected, including two ITCs, two CNs, and one EPN (**Figure 5**). A large proportion of GBPs were derived from sinigrin. The highest content of 2-propenyl isothiocyanate (SIN-ITC) was observed in the rhizomes of C1 grown in Leibo, and it was as high as 235.8 mmol kg^-1^. The content of SIN-ITC was also high in the roots (124.26 mmol kg^-1^); the content of SIN-ITC was lowest in the petioles (41.1 mmol kg^-1^), which was 17% of that in the rhizomes. The content of SIN-ITC was low in C1 grown in Guangyuan (average of 34.3 mmol kg^-1^). The content of SIN-ITC was high in the rhizomes and roots of C2 grown in Leibo (approximately 110 mmol kg^-1^); the content of SIN-ITC was highest in the rhizomes of C2 grown in Guangyuan (**Figure 5A**).

The content of 3-butenenitrile (SIN-CN) was highest in the rhizomes of C1 grown in Leibo, which was 4.3-fold higher than that in the leaves, which had the lowest content of SIN-CN. The SIN-CN content of the two cultivars grown in Guangyuan and C2 grown in Leibo was less than 2.0 mmol kg^-1^ (**Figure 5B**). 3,4-Epithiobutanenitrile (SIN-EPN) was the only EPN detected exclusively in the leaves, with the exception of C1 grown in Guangyuan, and the content of SIN-EPN was highest in C2 grown in Guangyuan (**Figure 5C**). 3-Butenyl isothiocyanate (GNA-ITC) was only detected in the rhizomes and roots. The content of GNA-ITC was highest in the rhizomes of C1 grown in Leibo. Variation in GNA-ITC among cultivars and producing areas was similar to that for SIN-ITC (**Figure 5D**). The content of 4-pentenenitrile (GNA-CN) was high in the rhizomes and roots in C1 from Leibo (1.8 and 1.4 mmol kg^-1^, respectively); the content of GNA-CN was highest in the rhizomes of C2 grown in Guangyuan, which was as high as 3.9 mmol kg^-1^ (**Figure 5E**). Variation in ITCs and GBPs was consistent with variation in SIN-ITC, while that of CNs was the superposition of those of SIN-CN and GNA-CN (**Figures 5F–H**).

### Correlation analysis

Correlation analysis of various phytochemicals provided various new insights (**Supplementary Table S2**). Strong positive correlations of pigments with soluble protein, total phenolics, and antioxidant capacity were observed. Specifically, the ABTS^+^ level was strongly correlated with the total phenolics content (0.987, *p*<0.01), and FRAP was strongly correlated with the ascorbic acid and total phenolic content (0.927 and 0.935, respectively, *p*<0.01). Glucosinolates were significantly and positively correlated with sinigrin, gluconapin, and progoitrin; GBPs were strongly correlated with SIN-ITC and GNA-ITC.

### Principal component analysis

**Figure 6:**
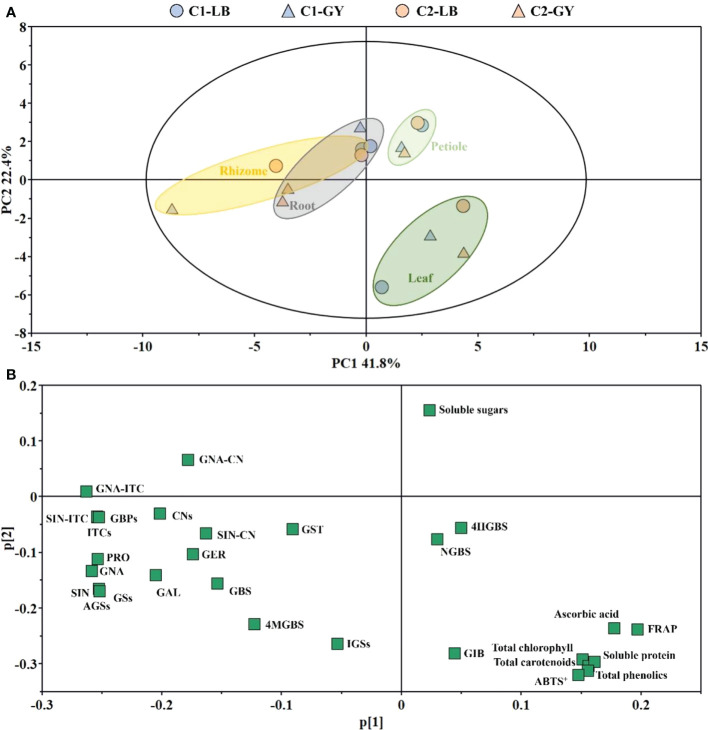
PCA in different organs of two cultivars of wasabi from different producing area. **(A)**: PCA score plot; **(B)**: PCA loading plot. C1, Chuankui–1; C2, Chuankui–2; LB, Leibo; GY, Guangyuan.

Variation in the main health-promoting compounds and antioxidant capacity among the different cultivars, producing areas, and organs was characterized by PCA (**Figure 6A**). The first principal component (PC1) and second principal component (PC2) explained 41.8% and 22.4% of the variance, respectively. Two groups could be discriminated along PC1 of the score plot: one group consisted of petioles and leaves, and the other group consisted of rhizomes and roots. Petioles could be distinguished from leaves by PC2. The loading plot indicated that the major contributors to the rhizomes and roots were GBPs and most glucosinolates, with the exception of 4-hydroxyglucobrassicin, neoglucobrassicin, and glucoiberin; soluble sugars were the major contributors to the petioles; and the major contributors to leaves were pigments, soluble protein, ascorbic acid, total phenolics, and antioxidant capacity (**Figure 6B**).

Next, OPLS-DA was performed to clarify variation among cultivars and producing areas (**Supplementary Figure S1**). The stability and reliability of the model are higher the closer the three indexes (R^2^X, R^2^Y, and Q^2^) are to 1. A model is considered effective when Q^2^ > 0.5. Producing areas (Q^2 =^ 0.849) were more distinguishable than cultivars. After the alignment verification of OPLS-DA (n = 200, that is, 200 permutation experiments were carried out), models were meaningful, and differential components can be screened based on variable importance plot (VIP) values. According to VIP values (>1) of the OPLS-DA, SIN-ITC, SIN-CN, GNA-ITC, ITCs, and GBPs contributed to variation among producing areas. Differences in C1 (Q^2^= 0.765) grown in Leibo and Guangyuan were more pronounced than differences in C2 grown in Leibo and Guangyuan (Q^2 =^ 0.209). In addition to the above-mentioned VIP values, CNs and GNA-CN also contributed to differences among groups (**Supplementary Table S3**).

### Variance analysis

The variance analysis revealed that cultivar, producing area, and the cultivar × producing area interaction affected the content of phytochemicals in each organ of wasabi (**Supplementary Table S4**). Cultivar and the cultivar × producing area interaction had stronger effects than producing area on the content of phytochemicals in the leaves; however, this was not the case for total chlorophyll, soluble protein, ABTS, glucoiberin, and 4-hydroxyglucobrassicin. Producing area had a stronger effect on all indicators in the petioles of wasabi, with the except of total carotenoids, soluble sugars, glucoiberin, 4-methoxyglucobrassicin, SIN-CN, GNA-CN, and CNs. The content of glucosinolates and GBPs was high in the rhizomes. Producing area and the cultivar × producing area interaction had greater effects than cultivar on the content of glucosinolates and GBPs. No differences in the relative importance of the different factors were observed for any indicators in the roots of wasabi.

## Discussion

Wasabi was introduced to mainland China in the early 1990s, and commercial production of wasabi was initiated in cool and humid mountains in Sichuan, Yunnan, Guizhou, and other provinces (Hao et al., 2016). Its rhizomes can be used in purees or processed into wasabi paste (Ravindran et al., 2012). In addition to being used in wasabi paste, leaves and petioles can be blanched and served cold or as suancai. In addition to differences in utilization methods, the dry matter content varies among organs, and this requires consideration when evaluating the nutrient benefits of the different organs.

Antioxidant activity was highest in wasabi leaves, regardless of cultivar and producing area. These data are consistent with the results of a previous study (Kinae et al., 2000; Jung et al., 2011; Shin et al., 2014; Hassan et al., 2021; Mitharwal et al., 2022). The leaves of taro (Colocasia *esculenta*) are rich in protein and have a balanced profile of amino acids and micronutrients, including bioactive chemicals (Mitharwal et al., 2022).Moringa *oleifera* leaves have received much attention interest in the fields of food and pharmaceutical development due to their high phenolics content (Hassan et al., 2021). The leaves are richer in phenolic compounds compared with the roots in sweet potato (Jung et al., 2011). In our study, the content of pigments and soluble protein was strongly correlated with antioxidant substances such as ascorbic acid and total phenolics as well as antioxidant capacity. This type of information can help consumers make purchasing decisions, especially for those that consider the nutrient content of products when buying food products (Di et al., 2022). FRAP was strongly positively correlated with ascorbic acid and total phenolics, indicating that FRAP is an overall indicator of the antioxidant capacity. ABTS was more strongly correlated with total phenolics than ascorbic acid, indicating that ABTS can be used as an indicator in phenolic-rich species such as Moringa *oleifera*. The total phenolics content and antioxidant capacity of Withania *somnifera* Dunal. were highest in the leaves at different growth stages, which indicated that total phenolics were positively related to antioxidant capacity (Fernando et al., 2013). The antioxidant activity and superoxide scavenging activity are higher in the leaves than in the roots of wasabi (Kinae et al., 2000). More detailed analyses of wasabi organs have shown that the antioxidant capacity is high in the leaves, fruit, and flowers but low in the petioles, rhizomes, and roots (Shin et al., 2014).

The soluble sugar content was higher in the petioles of wasabi than in other organs. Soluble sugars such as glucose and sucrose are the main photosynthetic products of plant photosynthesis, and they are also the main form of sugar used in carbohydrate metabolism and for temporary storage; they play key roles in the responses to various types of abiotic stresses through osmotic regulation (Du et al., 2020). For example, drought stress usually increases sucrose phosphate synthase activity, which may induce the accumulation of sucrose (Xu et al., 2015). In this study, the soluble sugar content was higher in the petioles than in the leaves, which is likely explained by the key role that petioles play in providing support to wasabi plants. Petioles can withstand greater stress than leaves, and this decreases their vulnerability to wilting and helps maintain the basic shape of plants. Another reason may be that sucrose is the main form of sugar for the long-distance transport of photosynthetic products (Yoon et al., 2021). The transport of sucrose from sources to sinks includes three steps: loading, transporting, and unloading. Herbaceous plants mainly load sucrose into the phloem (petioles) from sources (leaves) through active transport against the concentration gradient (Rennie and Turgeon, 2009). Due to the presence of a concentration gradient between petioles and sinks (rhizomes and roots), sucrose can continuously enter sinks to be utilized or stored. In addition, leaves are the main photosynthetic organs, and high concentrations of photosynthetic products inhibit photosynthesis in the leaves (Turgeon, 2010). A high soluble sugar content also improves the palatability and acceptability of suancai made from petiole–leaf mixtures, which has a sweet flesh and good mouth fragrance.

Rhizome is the most popular tissue used to make fresh pastes. It adds a unique flavour, heat, and greenery to food as a condiment and garnish (Depree et al., 1999). Sinigrin and SIN-ITC are rich in the rhizomes of wasabi and are described as having a sharp hot taste with a pungent smell, which leaves a pleasant mild vegetable flavour after the heat gradually dissipates in the mouth (Sultana et al., 2003a). The most abundant glucosinolates in wasabi along with sinigrin are endemic secondary metabolites of Cruciferae. In plants, the glucosinolate–myrosinase system, which is known as the “mustard oil bomb,” is a central component of the plant’s induced defense system (Miao et al., 2021). Glucosinolates and GBPs have been shown to protect plants from attack by insects and microorganisms (Zhang et al., 2022). This is also the reason for the high content of these compounds in rhizomes and roots compared with other organs. Previous studies have shown that the rhizomes of wasabi have the highest yield of ITCs compared with other organs, which makes them the most valuable (Sultana et al., 2003b). Similarly, the roots of radishes in the cruciferous family are rich in glucosinolates (32). The same is the case for most traditional medicines, in which the most common organs used are the rhizomes and roots. Ginsenosides, the main active components of Panax *ginseng* (P. *ginseng*), mainly exist in the rhizomes and roots of P. *ginseng* (Shi et al., 2007), American ginseng (Panax *quinquefolius*) (Qu et al., 2009), and Panax *notoginseng* (Tang et al., 2022); they have high medicinal value and differ in their functions. In humans, GBPs have also been identified as effective chemoprotectants against many chronic diseases such as cardiovascular disease and cancer (Miao et al., 2021). Sinigrin and SIN-ITC were the most abundant in wasabi, and these components have the greatest effect on flavour; 4-methoxyglucobrassicin is the glucosinolate with the strongest antibacterial effect among indole glucosinolates (Miao et al., 2021). Most GBPs are spontaneously formed ITCs. CNs and EPNs are generated by ESPs from glucosinolates under diverse conditions. SIN-EPN was only detected in the leaves, and GNA-CN was mainly present in the rhizomes and roots, which indicates the tissue specificity of environmental conditions such as pH and the presence of ferrous ions (Zhang et al., 2022).

Furthermore, the effects of cultivar and producing area on the nutritional components of various organs of wasabi were analyzed by variance analysis. Both leaves and rhizomes with higher utilization value were affected by the cultivar × producing area interaction. Differences in the annual average temperature, precipitation, and sunlight differed between the two sites. The average summer temperatures of the planting bases in Leibo and Guangyuan are 13 and 23°C, respectively, and the elevation of the Leibo planting base is nearly 900 m higher than that of Guangyuan; this is consistent with the cool growth requirements of wasabi and may contribute to the high quality of their rhizomes (Hodge, 1974). However, another study has shown that elevation had no significant effect on the content of ITCs, which might stem from the large differences between *Eutrema* species (Hao et al., 2016). The higher average winter temperature in Leibo (0.1°C) compared with Guangyuan (-6°C) provides some protection against the adverse effects of cold damage. Guangyuan is rainier than Leibo, and the annual rainfall at Guangyuan exceeds that of Leibo by more than 450 mm, especially in the summer; Guangyuan is thus hotter and more humid in the summer compared with Leibo. This may explain the higher glucosinolate content in the leaves of C1 planted in Guangyuan compared with Leibo, as these are produced to withstand the unfavorable growing conditions. The differences in the properties of the leaves and rhizomes of C1 between the two producing areas require consideration; rhizomes grown in Leibo were of better quality and can be directly sold or made into sauces, whereas the leaves of wasabi grown in Guangyuan could be used to produce suancai. In addition, the rhizomes of C2 grown in Guangyuan had more GBPs than C1 grown in Guangyuan, indicating that the two cultivars in the production areas differed.

## Conclusion

Differences in various chemical components of the organs of wasabi were studied, and the nutritional value of the leaves and petioles was explored. The content of pigments, soluble protein, ascorbic acid, and total phenolics, as well as the antioxidant capacity, was highest in the leaves; the content of soluble sugar was highest in the petioles. As expected, the rhizomes (except for C1 grown in Guangyuan) had the highest content of glucosinolates and GBPs. Producing area had a greater effect on the quality of wasabi than cultivar. In C1, rhizomes in Leibo and leaves in Guangyuan should be used reasonably according to local conditions. As far as rhizomes grown in Guangyuan are concerned, C2 is a better choice.

## Data availability statement

The original contributions presented in the study are included in the article/**Supplementary Material**. Further inquiries can be directed to the corresponding authors.

## Author contributions

HD: Investigation, Writing- Original draft preparation. CC: Data curation, Writing- Original draft preparation. PF: Investigation, Writing- Original draft preparation. JM: Data curation. MH: Investigation. ML: Data curation. WL: Investigation. FZ: Data curation. YZ: Funding acquisition, Writing- Reviewing and Editing, and Conceptualization. All authors: review and editing.

## Funding

This work was supported by the Sichuan Science and Technology Program (2021ZHFP0027), Central Guidance on Local Science and Technology Development Fund of Shaanxi Province (2022ZY1-CGZY-07), and Undergraduate Research Interest Cultivation Project of Sichuan Agricultural University (20220038).

## Conflict of interest

The authors declare that the research was conducted in the absence of any commercial or financial relationships that could be construed as a potential conflict of interest.

## Publisher’s note

All claims expressed in this article are solely those of the authors and do not necessarily represent those of their affiliated organizations, or those of the publisher, the editors and the reviewers. Any product that may be evaluated in this article, or claim that may be made by its manufacturer, is not guaranteed or endorsed by the publisher.
