# Supplementary material for: Variation in the main health-promoting compounds and antioxidant activity of different organs of Wasabi (Eutrema japonicum) from two producing areas
Source: Front Plant Sci. 2022 Oct 26;13:1043378. doi: 10.3389/fpls.2022.1043378 (PMC9643873; doi:10.3389/fpls.2022.1043378)

Supplementary Figure 1 Orthogonal partial least squares-discriminant analysis (OPLS-DA) of Leibo (LB) vs Guangyuan (GY) and Chuankui–1 grown in Leibo (C1-LB) vs Chuankui–1 grown in Guangyuan (C1-GY). R^2^X and R^2^Y represent the percentage of OPLS-DA model that can explain X and Y matrix information, respectively. Q^2^ is another important parameter for evaluating the OPLS-DA model.


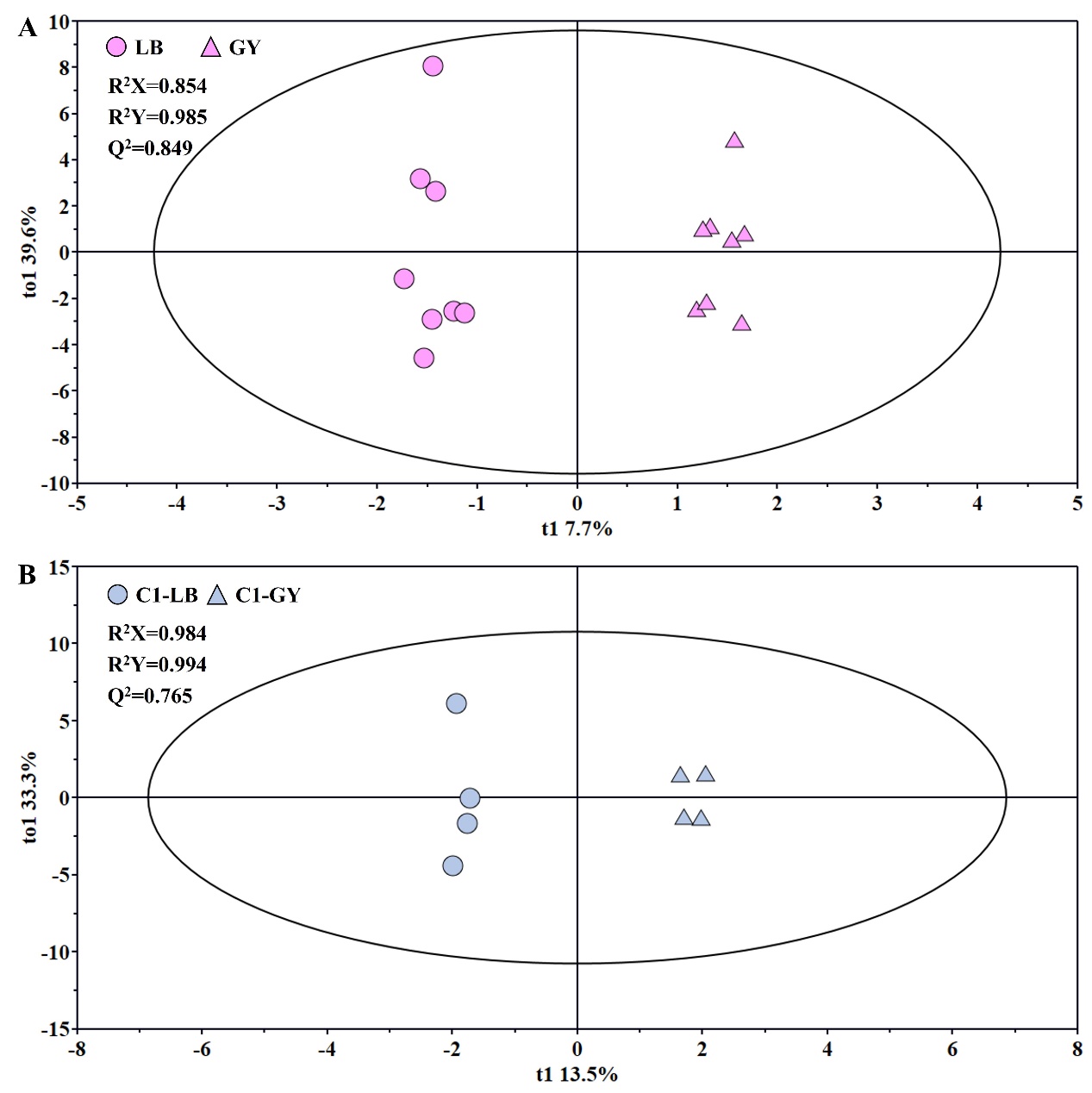

Supplement: Supplementary file 1 [file DataSheet_1.zip › Di et al. Supplementary Figure 1.docx]
